# Tilorone suppresses TGF-β–driven fibroblast activation and restores BMP–Smad1/5/8 signaling: implications for laryngotracheal fibrosis

**DOI:** 10.1186/s12967-026-08188-w

**Published:** 2026-05-09

**Authors:** Eniko Toth, Kitti Szabo, Attila Gergely Vegh, Agnes Zvara, Laszlo G. Puskas, Adam Bach, Ede Migh, Peter Horvath, Laszlo Tiszlavicz, Laszlo Rovo, Aniko Keller-Pinter

**Affiliations:** 1 Department of Biochemistry, Albert Szent-Gyorgyi Medical School, University of Szeged, Szeged, Hungary; 2 Centre of Excellence for Interdisciplinary Research, Development and Innovation, University of Szeged, Szeged, Hungary; 3 Institute of Biophysics, HUN-REN Biological Research Centre, Szeged, Hungary; 4 Laboratory of Functional Genomics, HUN-REN Biological Research Centre, Szeged, Hungary; 5 Department of Oto-Rhino‐Laryngology and Head-Neck Surgery, Albert Szent-Gyorgyi Medical School, University of Szeged, Szeged, Hungary; 6 Synthetic and Systems Biology Unit, HUN-REN Biological Research Centre, Szeged, Hungary; 7 Single-Cell Technologies Ltd, Szeged, Hungary; 8 Institute for Molecular Medicine, University of Helsinki, Helsinki, Finland; 9 Institute of AI for Health, Helmholtz Zentrum München, Neuherberg, Germany; 10 Department of Pathology, Albert Szent-Györgyi Medical School, University of Szeged, Szeged, Hungary; 11 Department of Internal Medicine, Albert Szent-Gyorgyi Medical School, University of Szeged, Szeged, Hungary

**Keywords:** Laryngotracheal fibrosis, Fibroblasts, Tilorone, α-smooth muscle actin, Bone morphogenetic protein, TGF-β, Collagen, Extracellular matrix stiffness

## Abstract

**Background:**

Laryngotracheal fibrosis is a rare but severe complication of prolonged intubation, leading to airway narrowing, respiratory distress, dysphonia, and, in advanced cases, life-threatening airway obstruction. Current treatments are primarily surgical, while pharmacologic approaches such as mitomycin C, corticosteroids, or 5-fluorouracil show inconsistent efficacy and potential toxicity. Thus, there remains a critical need for safe and effective antifibrotic therapies. Transforming growth factor-beta (TGF-β) is a key mediator of fibrosis, promoting fibroblast activation, migration, and expression of profibrotic markers such as alpha-smooth muscle actin (α-SMA).

**Objectives:**

This study aimed to evaluate the antifibrotic potential of tilorone dihydrochloride, a synthetic small molecule, in human respiratory fibroblasts in vitro.

**Methods:**

Fibrotic alterations were assessed in human laryngotracheal fibrosis tissue samples. An in vitro model using MRC-5 human lung-derived fibroblasts was employed to investigate the effects of tilorone. Molecular analyses (RT-qPCR, immunocytochemistry, western blotting) quantified mRNA and protein expression of key signaling markers. Cell proliferation and viability were performed to evaluate potential cytotoxic effects of tilorone. Functional assays, including wound scratch and single-cell tracking, assessed fibroblast motility, and atomic force microscopy (AFM) measured extracellular matrix elasticity.

**Results:**

Phosphorylation of Smad1/5/8, a key transcription factor in the bone morphogenetic protein (BMP) signaling, was reduced in both human laryngotracheal fibrosis tissue and TGF-β-treated MRC-5 fibroblasts. Tilorone treatment did not affect MRC-5 cell viability or proliferation but increased BMP2, BMP4, BMP7, and BMP14 (GDF5; Growth Differentiation Factor 5) mRNA expression, enhanced Smad1/5/8 phosphorylation, and suppressed TGF-β-induced Smad2/3 phosphorylation. Functionally, tilorone inhibited TGF-β-driven fibroblast migration and α-SMA expression, downregulated collagen I and III mRNA levels, and restored extracellular matrix elasticity, as confirmed by AFM.

**Conclusion:**

Tilorone counteracts TGF-β-mediated profibrotic signaling and restores BMP pathway activity in MRC-5 fibroblasts in vitro. These findings identify tilorone as a promising therapeutic candidate for fibrotic airway diseases such as laryngotracheal fibrosis.

**Supplementary Information:**

The online version contains supplementary material available at https://doi.org/10.1186/s12967-026-08188-w.

## Introduction

Fibrosis is characterized by the excessive accumulation of extracellular matrix (ECM), which can lead to the distortion of normal tissue architecture and progressive loss of organ function. This pathological process is primarily driven by elevated levels of profibrotic cytokines, particularly transforming growth factor-beta (TGF-β), which are abundantly secreted during wound healing and tissue repair processes [1, 2]. Although fibrosis commonly affects organs such as the lungs, heart, kidneys, and liver, emerging evidence points to an increasing prevalence of fibrotic complications in less typical sites, including the trachea and larynx. Approximately 35.4% of deaths in the developed world are associated with chronic fibroproliferative diseases [3], highlighting the urgent need for effective antifibrotic therapies across diverse tissues and clinical contexts.

Laryngotracheal stenosis is the result of abnormal and excessive wound healing that produces hypertrophic scarring and narrowing of the upper airway. This condition can affect the glottis, supraglottis, subglottis, or trachea and may result in severe respiratory compromise, including life-threatening airway obstruction. It most frequently develops after airway interventions, particularly endotracheal intubation and tracheostomy [4, 5]. Prolonged intubation can cause localized ischemia due to sustained cuff pressure on the tracheal wall, initiating a cascade of tissue damage and maladaptive healing [6]. Although patients may initially appear asymptomatic post-extubation, fibrotic web-like tissue can form within 3 to 6 weeks [6, 7], leading to post-intubation laryngotracheal stenosis. Reported incidence rates vary widely: in a prospective cohort of 100 patients, 57% demonstrated acute laryngeal injury, including ulceration or granulation tissue formation [8]; in a retrospective analysis of 1,130 tracheostomies, the incidence was 1.9% [9]; while other reviews estimate a broader range, with a mean incidence of 8.9% (range: 0%–20.8%) [10]. Pediatric cases also show concerning trends, with an 11.38% incidence of subglottic stenosis [11]. Although various pharmacologic agents, such as mitomycin C [12, 13], corticosteroids [14, 15], and 5-fluorouracil [16], have been explored as adjunctive treatments for laryngotracheal fibrosis, their clinical outcomes remain inconsistent. They may transiently reduce fibrotic tissue or delay restenosis, but their efficacy is variable and often limited by impaired wound healing or toxicity. Consequently, current management still relies predominantly on surgical intervention, highlighting the need for safer and more effective antifibrotic strategies.

TGF-β and bone morphogenetic proteins (BMPs) are both members of the transforming growth factor superfamily and play crucial roles in maintaining tissue homeostasis and facilitating regeneration following injury [17, 18]. However, dysregulated or excessive TGF-β signaling leads to the activation of numerous profibrotic genes, including those encoding growth factors, ECM components, adhesion molecules, and other elements of the TGF-β signaling network. In contrast, BMP signaling, particularly via BMP2, BMP4, and BMP7, exerts antifibrotic effects by counteracting TGF-β activity and modulating epithelial–mesenchymal transition [19–24]. Notably, exogenous administration of BMP7 has been shown to reduce experimentally induced fibrosis in multiple organs, including the kidney, heart, liver, lung, and eye.

Tilorone dihydrochloride was first described in the 1970s as an interferon-inducing agent [25, 26]. Since then, several studies have explored its mechanisms of action and broad therapeutic potential. Its antifibrotic effects were initially reported in pulmonary fibrosis, where tilorone enhanced BMP and interferon signaling, inhibited TGF-β activity, and reduced collagen expression in lung endothelial cells [27]. Subsequent studies confirmed its efficacy in preventing lung fibrosis in a mouse model using an inhaled tilorone formulation [28], and, more recently, demonstrated its ability to reverse myocardial fibrosis in vivo [29].

The global incidence of fibrosis and its associated health problems is increasing, and fibrosis is recognized as one of the major health challenges [1, 11]. In this study, we observed reduced phosphorylation of Smad1/5/8—a key BMP signaling effector—in fibrotic human laryngotracheal tissue and TGF-β-treated MRC-5 fibroblasts. Because tilorone has been shown to enhance BMP signaling in epithelial cells [27], myoblasts [30], and steatotic liver tissue samples [31], we hypothesized that it could attenuate laryngotracheal fibrosis by promoting BMP-Smad1/5/8 signaling.

In our study, tilorone increased cellular levels of phosphorylated Smad1/5/8, a key signaling molecule in the BMP pathway, and upregulated BMP expression. Furthermore, tilorone significantly decreased TGF-β-induced Smad2/3 phosphorylation, fibroblast migration, and alpha-smooth muscle actin (α-SMA) expression, a hallmark of myofibroblast differentiation. Tilorone also downregulated TGF-β-induced transcription of matrix-forming proteins, including collagen types I and III, and preserved extracellular matrix mechanical properies (elasticity). Together, these data identify tilorone as a promising antifibrotic candidate for airway-associated scarring disorders such as laryngotracheal fibrosis and represent an early step in translating mechanistic discoveries into therapeutic innovation.

## Methods

### Human laryngotracheal tissue samples

Human laryngotracheal fibrous tissue samples were collected from patients suffering from laryngotracheal stenosis resulting from prolonged intubation. The histological samples were obtained during airway-widening surgery performed via an external approach. In one patient, an open airway procedure had been performed prior to the current surgery, while in three patients, previous endoscopic airway surgeries (laser resection, coblation, stent placement) had been carried out earlier. In the remaining three patients, tracheotomy had been performed before the current intervention. The fibrotic airway segment was excised using cold instruments and fixed in 4% formalin. Ethical approval for the study was obtained from the Human Institutional and Regional Biomedical Research Ethics Committee, University of Szeged (100/2022-SZTE RKEB). All methods were performed in accordance with the relevant guidelines and regulations, and informed consent was obtained from all subjects and/or their legal guardian(s).

### Immunohistochemistry

Human laryngotracheal tissue samples were fixed in 4% formalin, and embedded in paraffin, then sections were stained with α-SMA (202 M-94; Cell Marque Corporation, Rocklin, California, USA), desmin (IR606; DAKO, Glostrup, Denmark), vimentin (NCL-L-VIM-V9; Novocastra-Leica; Deer Park, IL, United States) and pSmad1/5/8 (Ser463/465) (AB3848-I; Abcam, Cambridge, United Kingdom) primary antibody, followed by the incubation with the appropriate peroxidase-conjugated secondary antibody. The nuclei were stained with hematoxylin, and the immunocomplexes were visualized with 3,3’-diaminobenzidine. High-resolution fluorescence images were acquired using a Zeiss Imager Z1 microscope with a 20× objective (Zeiss Plan Neoflux 20 ×). Representative images of fibrotic and non-fibrotic tissues derived from the same patient, and within each sample, identical microscopic areas were analyzed across serial sections stained for α-SMA, vimentin, and desmin. Nuclear pSmad1/5/8 levels were measured using QuPath-0.5.0 and ImageJ Software.

### Cell cultures

MRC-5 human fetal lung-derived fibroblasts (ATCC, CCL-171) were cultured in 90% Eagle’s Minimum Essential Medium (EMEM; 10-009-CV, Corning, NY, USA) supplemented with 10% fetal bovine serum (FBS; Gibco, Life Technologies, Waltham, MA, USA) and 65 mg/mL gentamicin (Sandos, Basel, Switzerland). Cells were treated with TGF-beta (TGF-β; 5 ng/mL, PHP143B, Bio-Rad, Hercules, California, USA) and 2,7-bis[2-(diethylamino)ethoxy]-9-fluorenone dihydrochloride (tilorone dihydrochloride; #220957, Sigma-Aldrich, St. Louis, MO, USA) at a concentration of 20 or 35 nM.

### Cell proliferation and viability

Cell proliferation and viability were assessed by automated cell counting using a Luna 3 cell counter (Logos Biosystems, Anyang-si, Gyeonggi-do, South Korea). Cells were stained with erythrosin B (#189460250, Thermo Fisher Scientific, Waltham, Massachusetts, USA), and viable and non-viable cells were distinguished based on dye exclusion. Measurements were performed at 0, 24, 48, and 72 h following treatment with 10, 20, 35, 50, and 100 nM tilorone. To assess cell proliferation, the number of viable cells was normalized to the 0-hour time point. Cell viability was determined as the ratio of viable to total cell numbers.

### Real-time quantitative PCR

The treatment was performed on cell cultures seeded in a 6 cm diameter cell-culture dish at a density of 360,000 cells/well. Cells were treated for 72 h in a medium supplemented with TGF-β, TGF-β + 20 nM tilorone, or TGF-β + 35 nM tilorone. At the end of the treatment periods, cells were lysed with 600 µl RA1 / β-mercaptoethanol lysis buffer and prepared for qPCR assay. The qRT-PCR experiments were performed using a RotorGene 3000 machine (Qiagen, Hilden, Germany), gene expression was analyzed using gene-specific primers and SybrGreen protocol. One µg total RNA was reverse transcribed in 30 µl final volume using the High-Capacity cDNA Archive Kit (Thermo Fisher Scientific) according to the manufacturer’s instructions.

The reverse transcription protocol was as follows: samples were incubated for 10 min at room temperature, 2 h at 37 ℃, 5 min on ice, and then 5 min at 85℃ to inactivate the enzyme. After 3 × dilution, 1 µl of diluted reaction mix was used as a template for qPCR. The reactions were performed using qPCRBIO SyGreen Lo-ROX mix (PCR Biosystems, London, UK) according to the manufacturer’s instructions at a final primer concentration of 250 nM, with the following cycle: 2 min at 95 °C, 40 × 5 s at 95 °C, followed by 30 s at 60 °C. After each reaction, the melting temperature was measured to check the quality of the products. Primers were designed using the Roche Universal Probe Library Assay Design Center online program. The quality of primers was checked by MS analysis by Bioneer (Daejeon, South Korea). Individual threshold cycle (Ct) values were normalized to the average Ct values of glyceraldehyde-3-phosphate dehydrogenase (GAPDH), glucuronidase beta (GUSB), and ribosomal protein lateral stalk subunit P0 (RPLP0) as internal control genes. Relative gene expression levels were calculated using 2^−ΔΔCt^ ratios. Information on genes and primers used is provided in Table 1.

**Table 1:**
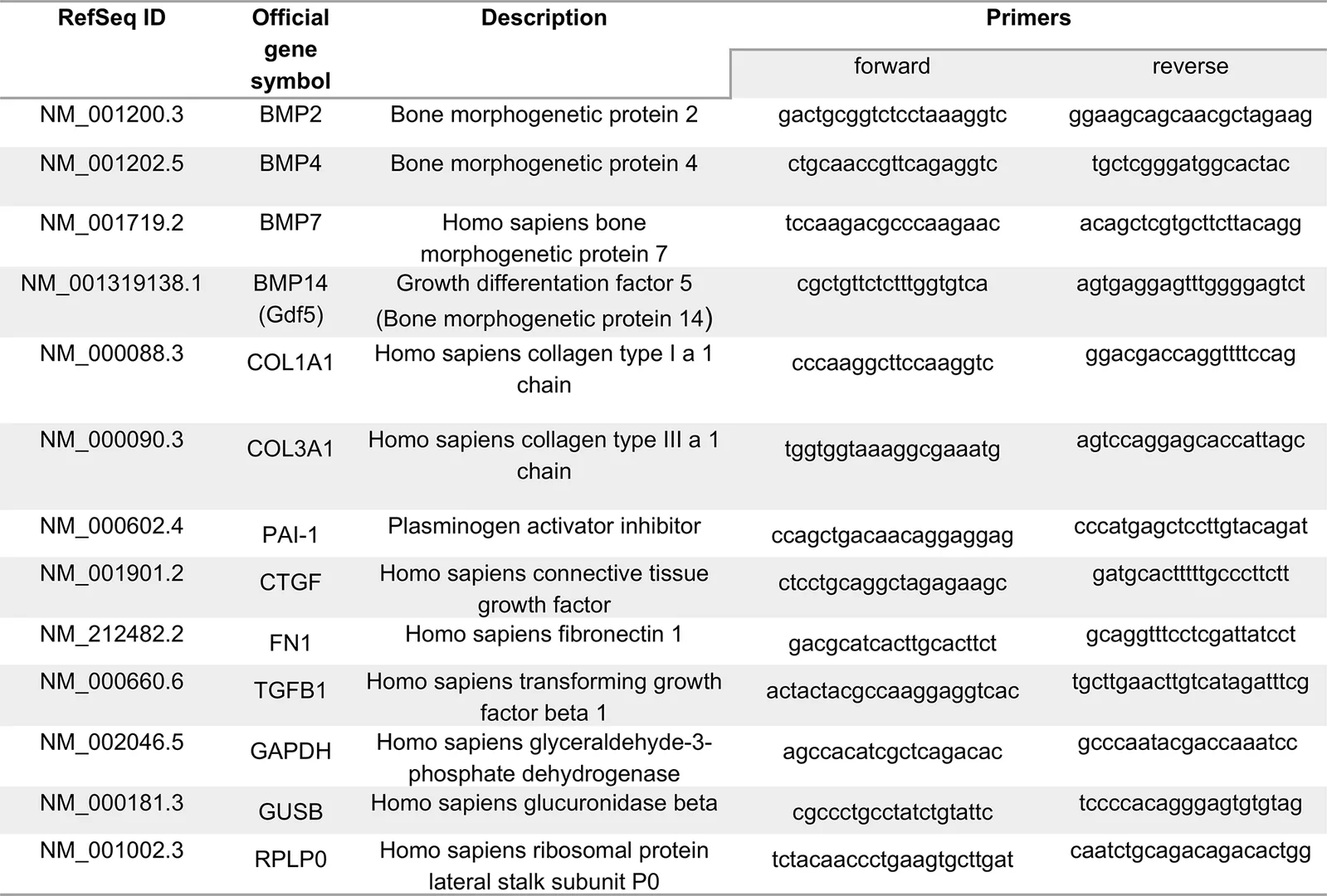
List of investigated genes and primers used in qRT-PCR experiments

### Immunoblotting

MRC-5 cells were lysed in RIPA buffer (20 mM Tris-HCl pH 7.5, 150 mM NaCl, 1 mM Na_2_EDTA, 1 mM EGTA, 1% NP-40, 1% sodium deoxycholate, 2.5 mM sodium pyrophosphate, 1 mM β-glycerophosphate, 1 mM Na_3_VO_4_, 1 µg/mL leupeptin; #9806; Cell Signaling Technology, Danvers, Massachusetts, USA) supplemented with 1 mM sodium fluoride and a protease inhibitor cocktail (#P8340; Sigma-Aldrich). First, the lysates were centrifuged at 16,000×*g* for 5 min at 4 °C, and then protein levels were determined using the bicinchoninic acid method (#23227; Thermo Fisher Scientific). Equal amounts of proteins were separated via sodium dodecyl sulfate-polyacrylamide gel electrophoresis and transferred to Protran nitrocellulose membrane (GE Healthcare). The membranes were blocked in EveryBlot Blocking Buffer (#12010020; Bio-Rad Laboratories, Inc., Hercules, California, USA) for 5 min at room temperature. Then, the membranes were incubated at 4 °C overnight with the following rabbit polyclonal primary antibodies: pSmad1/5/8 (NB 100-56656, Novus Biologicals, Centennial, Colorado, USA), Smad1/5/8 (AB-3848-I; Sigma-Aldrich), pSmad2/3 (PA5-110155; Sigma-Aldrich), Smad2/3 (#8655; Cell Signaling Technology), Phospho-p38 MAPK (Thr180/Tyr182) (#28796-1-AP; Proteintech, Rosemont, Illinois, USA), p38 MAPK (#14064-1-AP; Proteintech), α-SMA (#1925; Cell Signaling Technology), and GAPDH (#2118; Cell Signaling Technology). Membranes were subsequently incubated with the horseradish peroxidase-conjugated anti-rabbit IgG secondary antibody (#P0448) from DAKO. Peroxidase activity was developed using the enhanced chemiluminescence (K-12045 Advansta, San Jose, California, USA) procedure, and the chemiluminescence signal was recorded using X-ray films (Agfa). On the scanned X-ray films, bands were selected with uniform square ROIs (region of interest) using the BioRad Quantity One Analysis Software (Bio-Rad). Then, using the “Volume Analysis Report” function, we obtained the intensity of the selected area normalized to the background.

### Fluorescence immunocytochemistry and microscopy

To assess the nuclear phospho-Smad1/5/8 levels, cells were grown in a 6-well cell culture dish at a density of 180,000 cells/22 × 22 cm coverslip, then fixed on day 3 with 4% paraformaldehyde. After washing in PBS for 3 × 5 min and permeabilization with 0,2% Triton X-100 diluted in PBS for 10 min, samples were blocked with 4% BSA (bovine serum albumin) in PBS for 1 h. After washing with PBS 3 × 5 min, the cells were incubated with pSMAD1/5/8 (1:500, rabbit, #13820S, Cell Signalling Technology, Danvers, Massachusetts, USA) primary antibody overnight at 4 °C. The next day, cells were incubated with Alexa488-conjugated anti-mouse antibody (1:100, 715-545-151, Jackson Immunoresearch, West Grove, Pennsylvania, USA) at room temperature in the dark for 60 min. Nuclei were stained with Hoescht 33258 (0.01 mg/mL, Sigma-Aldrich, Darmstadt, Germany) for 5 min. Fluorescent Mounting Medium (10068918, DAKO, Glostrup, Denmark) was used to cover the sample.

To visualize α-SMA expression, cells were grown in 6-well cell culture dish at a density of 180,000 cells/22 × 22 cm coverslip. After 4 days, cells were fixed with 4% paraformaldehyde and then washed with 0,1% Tween 20 dissolved in 4% PBS, followed by permeabilization with 0,5% Triton X-100 diluted in PBS for 20 min. Then, samples were blocked with 3% BSA in PBS for 1 h and immediately incubated with primary antibody to α-SMA (1:50, D4K9N, Cell Signalling Technology) overnight at 4 °C. The next day, cells were washed with PBS for 3 × 5 min and 0.1% Tween 20 in PBS for 1 × 5 min, and incubated with Alexa488-conjugated anti-mouse antibody (1:100, 715-545-151, Jackson Immunoresearch, West Grove, Pennsylvania, USA) in the dark at room temperature for 60 min. After 4 × 5 min washing in PBS and 1 × 5 min washing in 0.1% Tween 20 in PBS, nuclei were stained with Hoescht 33258 for 10 min, and samples were mounted with. ProLong Gold (9071S, Cell Signaling Technology).

High-resolution fluorescence images were acquired using a Nikon Eclipse Ni-U fluorescence microscope (Nikon Instruments Inc., Melville, NY, USA) with a 100× objective (Nikon Plan Apo 100 ×/1.45 oil, DIC N2) and analyzed using EpiSelector image analysis software. A total of 20–25 fields of view per cell line were analyzed per independent experiment.

### Wound scratch assay

Cells were seeded onto coverslips in 6-well plates at a density of 3 × 10^5^ cells per well. After a 12-hour incubation in a proliferating medium, the cells were incubated for 24 h in a serum-reduced medium with treatment to inhibit cell division. A cell-free zone (wound scratch) was created manually using a 200 µl pipette tip. Wound closure was monitored every 4 h over a 12-hour period using a 10× objective on a Leica DM3000 microscope (Leica, Wetzlar, Germany). The area of the cell-free zone was quantified using Digimizer image analysis software (MedCalc Software bvba, Ostend, Belgium). Wound closure was calculated as: (area of cell-free zone at 0 h - area of cell-free zone at x h) / area of cell-free zone at 0 h.

### Time-lapse imaging of live cells

To analyse random cell migration, live-cell imaging was performed with the Operetta (PerkinElmer, Inc., Waltham, MA, USA) high-content imaging system, utilizing a 20× objective (20× long WD; NA = 0.45, working distance: 7.8 mm; field of view: 675 × 509; depth of focus: 4.6 mm; optical xy resolution: 0.7 mm). For imaging, cells were seeded in 24-well plates (Thermo Fisher Scientific) at a density of 10^4^ cells/well. TGF-β and TGF-β + 20 nM tilorone cotreatment in serum-reduced medium (98% EMEM + 2% FBS) lasted for 90 h, including the 18 h incubation in the microscope. Before imaging, the nuclei were stained with Hoechst 33342 (1:2000, 1 mg/mL stock solution; Sigma-Aldrich). Time-lapse images were obtained automatically at 20 min intervals for 18 h at 37 °C and 5% CO2.

### Single-cell tracking of cultured fibroblasts

Time-lapse microscopic images were analyzed using ImageJ (National Institutes of Health, Bethesda, MD, USA, https://imagej.nih.gov/ij/) and CellTracker (http://celltracker.website/) software programs. The nuclei were tracked manually through every frame, and the x and y coordinates of the movement were recorded. We excluded dying, dividing, or damaged cells from the analysis. Based on the nuclear coordinates, the length of the total path, the maximal distance from the origin, and the average speed were calculated.

To visualize the trajectories, the migratory paths of individual cells were aligned to a common origin using the Excel DiPer Plot_At_Origin macro [32]. Wind rose plots were generated to visualize each cell’s movement, with trajectories derived from the x- and y-coordinate data obtained through cell tracking.

### Atomic force microscopy

Experiments were carried out with an NTEGRA Spectra II (NT-MDT, Spectrum Instruments) atomic force microscope and controller, using driving software NOVA Px (version 3.4.1). For matrix elasticity measurements, BL-RC150VB (Olympus Co. Japan) short levers with a V-shaped tip were used. The spring constant of the cantilever was calibrated each time prior to experiments by built-in calibration methods [33, 34]. All maps were recorded in fresh cell-growth media. For each sample, at least three distantly located areas of 40 μm by 10 μm were scanned using the HybriD mode of the system – an oscillatory non-resonant mode, in which the probe and the sample are brought into intermittent contact in the vertical direction and the induced probe deflection is employed for surface tracking feedback.

Selected intercellular areas were divided into 256 by 64 pixels, and a force-distance curve was recorded for each pixel, applying a vertical tip-traveling distance of 200 nm and a maximal indenting force of 400 pN at a frequency of 600 Hz. During the raster scan, feedback was used on the maximal indenting force. Both trace and retrace maps were recorded separately and compared, keeping only those for further analysis where no considerable differences were observed between the two.

For quantification of the results, the recorded force volume maps were analyzed with the driving software of the system, calculating the sample’s elastic modulus [35] for each force curve. This resulted in elasticity maps and visualization of the results was performed using a custom-written MATLAB (Mathworks, R2015b) routine. The logarithm of the calculated elastic modulus is normally distributed, which facilitates the comparison between the investigated samples. Higher elastic values are associated with more rigid materials, while lower values are associated with softer materials.

### Statistical analysis

Statistical analyses were performed using GraphPad Prism 7 software (GraphPad Software Inc., San Diego, CA, USA), one-way ANOVA, and post hoc test (Holm-Sidak) for pairwise comparisons of groups. All evaluated data were expressed as mean + SEM. Individual p-values were considered significant at *p* < 0.05. Individual p-values and significance levels are indicated on graphs.

## Results

### Activated fibroblasts exhibit low phospho-Smad1/5/8 levels in human laryngotracheal tissue samples

Smad1/5/8 proteins are key intracellular mediators of BMP signaling. Upon BMP ligand binding to its transmembrane receptor, Smad1/5/8 becomes phosphorylated and translocates to the nucleus to mediate transcriptional responses [36]. Numerous studies have already demonstrated that fibrosis is associated with reduced levels of BMPs and decreased phosphorylation of Smad1/5/8, largely due to antagonism by the TGF-β signaling pathway [37, 38]. To investigate this phenomenon in laryngotracheal fibrosis, we analysed histological samples from cases of human laryngotracheal fibrosis.

**Fig. 1:**
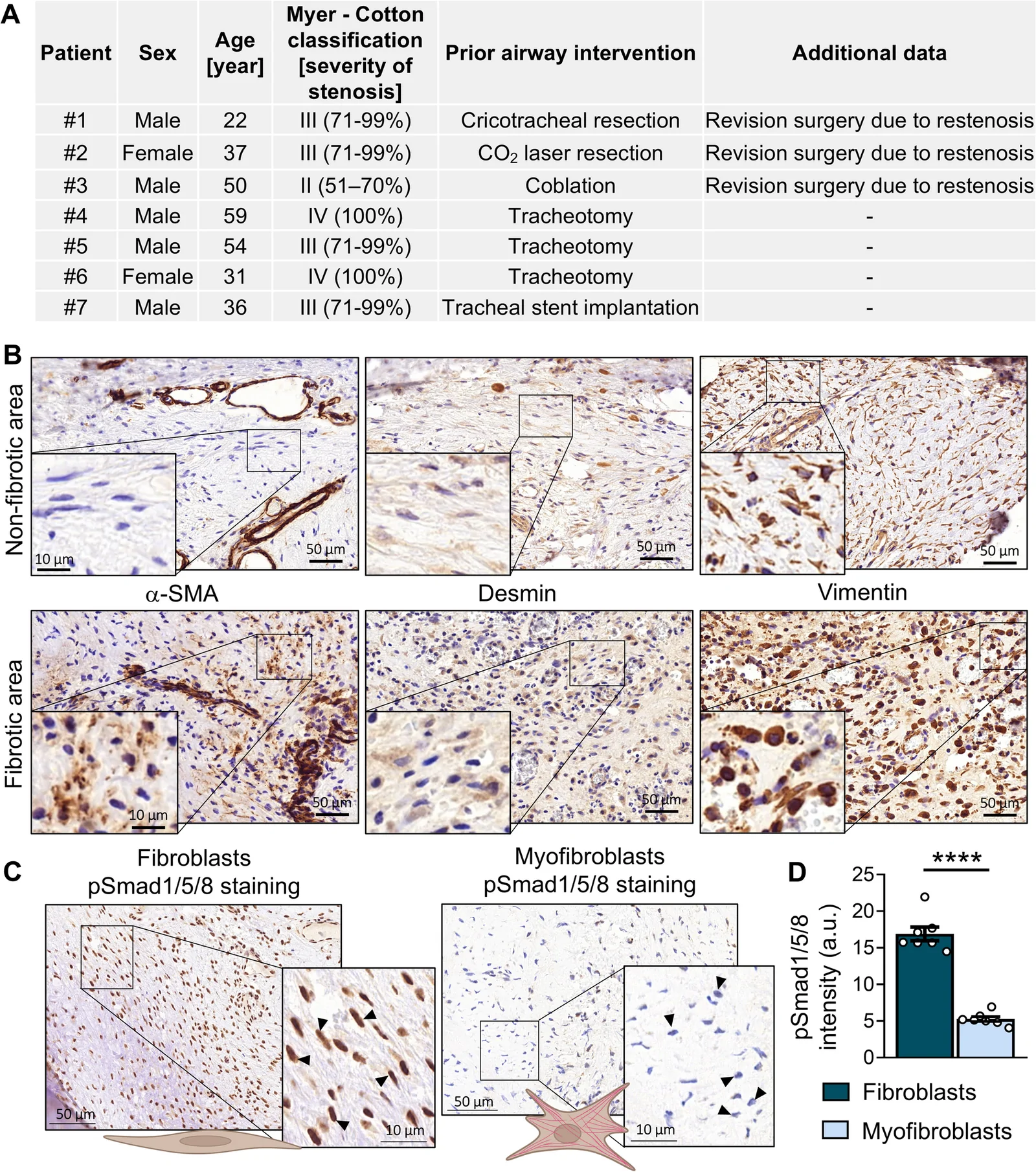
Activated fibroblasts in human laryngotracheal tissue samples exhibit low phospho-Smad1/5/8 levels. **A** Summary of patient data of human tissue samples with laryngotracheal fibrosis. Subglottic stenosis was graded according to the Myer–Cotton classification as follows: Grade I (0–50% stenosis), Grade II (51–70% stenosis), Grade III (71–99% stenosis), and Grade IV (100% stenosis). **B** Representative immunohistochemical staining for α-SMA, desmin, and vimentin to distinguish fibroblasts (α-SMA⁻/desmin⁻/vimentin⁺) and myofibroblasts (α-SMA⁺/desmin⁻/vimentin⁺) in the non-fibrotic and fibrotic regions of human laryngotracheal tissue samples. **C** Representative histological images show differences in nuclear pSmad1/5/8 staining between fibroblasts and myofibroblasts. Brown: pSmad1/5/8, blue: hematoxylin-stained nuclei. **D** Quantification of pSmad1/5/8 intensity (*n* = 7 independent human samples; 15 cells/sample). Data are reported as the mean ± SEM; a.u.: arbitrary unit, ****p < 0.0001

Histological specimens were obtained during open-airway reconstructive procedures performed several months after prolonged intubation. At the time of sampling, the stenotic lesions were considered permanent, and the associated scar tissue was classified as mature. The severity of subglottic stenosis was graded (Fig. 1A) according to the Myer–Cotton classification as follows: Grade I (0–50% stenosis), Grade II (51–70% stenosis), Grade III (71–99% stenosis), and Grade IV (100% stenosis) [39]. All patients had undergone previous airway interventions, such as tracheotomy, stenting, endoscopic or open surgical procedures, prior to the definitive external airway reconstruction (Fig. 1A).

To distinguish fibrocytes from their activated form, myofibroblasts - hallmarks of fibrotic tissue - we performed immunohistochemical staining for α-SMA, desmin, and vimentin on histological sections (Fig. 1B). α-SMA was used to identify activated myofibroblasts associated with fibrotic remodeling, whereas vimentin served as a general mesenchymal marker for stromal fibroblasts. Desmin, a muscle-specific intermediate filament protein, was included to distinguish muscle cells from fibroblast-derived myofibroblasts. The absence of desmin staining in both fibrotic and non-fibrotic regions indicates that the analyzed areas consisted predominantly of stromal rather than muscle tissue. Collectively, the expression pattern of these markers in the non-fibrotic and fibrotic regions supports the identification of fibroblasts (α-SMA⁻/desmin⁻/vimentin⁺) and myofibroblasts (α-SMA⁺/desmin^-^/vimentin⁺), complementing morphological characteristics.

Fibrotic and non-fibrotic subareas were identified and analyzed within each patient sample. Control fibroblasts in non-fibrotic tissue exhibit strong nuclear pSmad1/5/8 immunoreactivity, whereas fibrosis-associated myofibroblasts in fibrotic regions show markedly reduced pSmad1/5/8 levels (Fig. 1C). Quantitative image analysis of histological sections confirmed a significant decrease in nuclear pSmad1/5/8 intensity in myofibroblasts (Fig. 1D).

### Tilorone does not affect fibroblast proliferation or viability

**Fig. 2:**
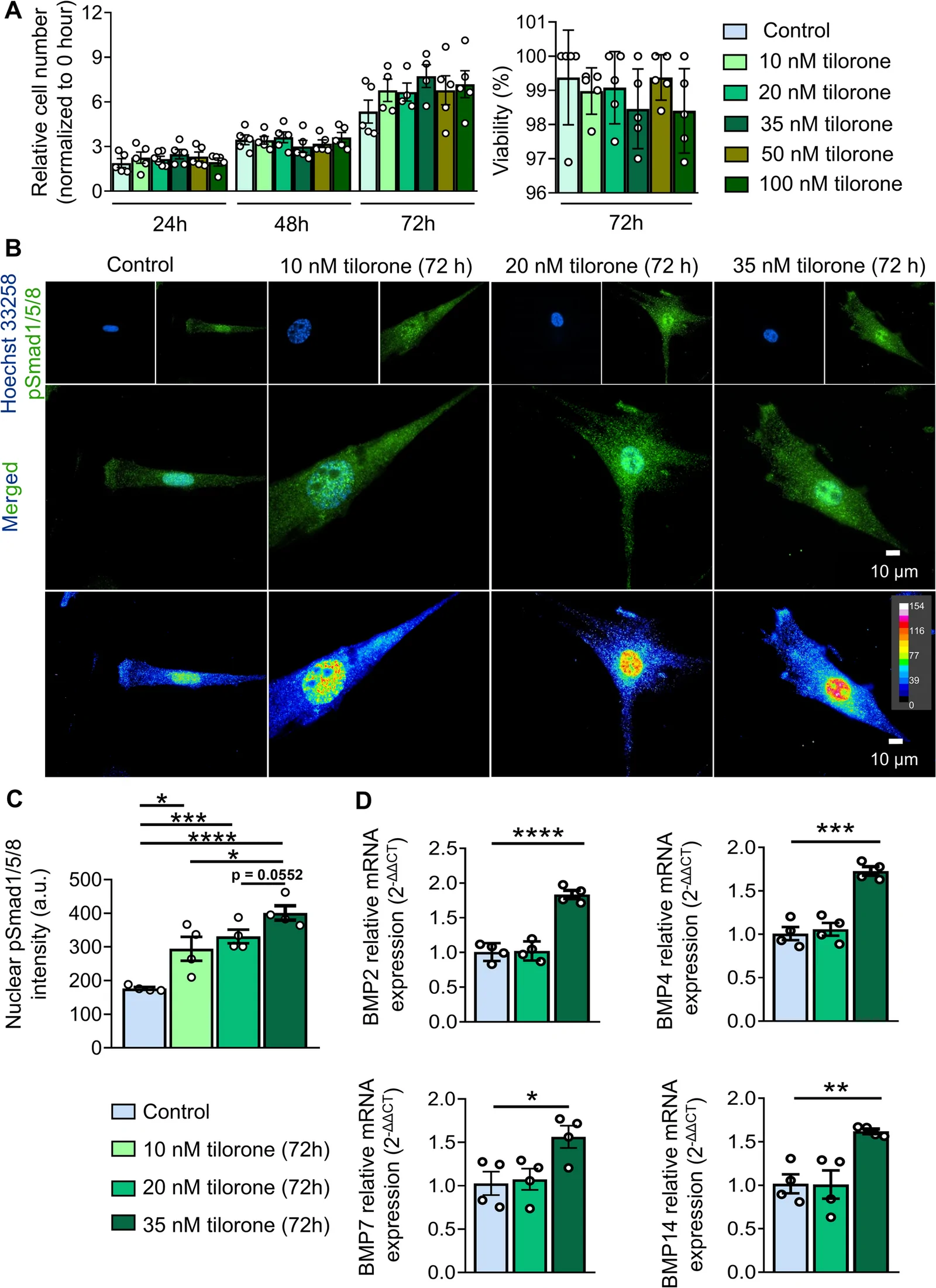
Tilorone increases nuclear phospho-Smad1/5/8 levels in vitro and induces bone morphogenic protein (BMP) expression without affecting fibroblast proliferation or viability. **A** Proliferation and viability of MRC-5 fibroblasts were assessed at different tilorone concentrations (0, 10, 20, 35, 50, 100 nM; *n* = 5 independent experiments). Cell proliferation was measured at 24, 48, and 72 h after tilorone treatment. Cell viability results are shown after 72 h tilorone treatment. **B** Representative wide-field fluorescence images reveal the nuclear distribution of pSmad1/5/8 following staining with Alexa Fluor 488 (green) of control and tilorone treated (10 nM, 20 nM, or 35 nM for 72 h) cells. Nuclei were stained by Hoechst 33258 (blue). Pseudo-color images depict pSmad1/5/8 nuclear intensity as indicated by the calibration bar. **C** The mean intensity values of the cells were quantified, normalized to control, and compared following treatment (*n* = 4 independent experiments, 20-25 cells/treatment were quantified). **D** MRC-5 fibroblasts were treated with 35 nM tilorone for 24 and 72 h, and the gene expressions of BMP2, BMP4, BMP7, and BMP14 were analyzed by quantitative real-time PCR (*n* = 4 independent experiments). Data are reported as the mean ± SEM; a.u.: arbitrary unit; *p < 0.05; **p < 0.01; ***p < 0.001; ****p < 0.0001)

Since we observed alterations in Smad1/5/8 signaling between fibroblasts and myofibroblasts in human tissue samples, next we aimed to investigate the effect of tilorone on MRC5 fibroblasts, a cell line previously utilized in studies of laryngeal fibrosis [13]. We first assessed whether tilorone influences fibroblast proliferation or survival. For that, we conducted a time-course proliferation assay and erythrosin B exclusion–based viability analysis. Live, dead, and total cell numbers were recorded at 0, 24, 48, and 72 h following treatment. Tilorone did not alter proliferation of MRC-5 fibroblasts at any tested concentration. Moreover, cell viability remained between 95 and 100% throughout the 72-hour treatment period, indicating the absence of cytotoxic effects (Fig. 2A). These findings demonstrate that tilorone does not impair fibroblast proliferation or survival under the experimental conditions applied.

### Tilorone increases bone morphogenetic protein (BMP) expression and phospho-Smad1/5/8 levels in MRC-5 cells

To assess whether tilorone activates the BMP-pSmad1/5/8 signaling pathway in fibroblasts, we performed immunocytochemical analysis of phosphorylated Smad1/5/8 (pSmad1/5/8) in MRC5 fibroblasts following treatment with increasing concentrations of tilorone (10, 20, and 35 nM) (Fig. 2B). The tilorone concentration and treatment durations were selected based on previous studies of Kohler et al. [30]. The quantified results demonstrated a dose-dependent increase in nuclear pSmad1/5/8 localization (Fig. 2C), confirming BMP pathway activation in fibroblasts in vitro.

Tilorone has been reported to enhance BMP expression in epithelial cells, thereby activating the BMP signaling pathway and antagonizing the profibrotic TGF-β pathway [27]. We further demonstrated this BMP-inducing effect of tilorone in myoblasts [30]. Next, we aimed to investigate whether tilorone similarly upregulates BMP expression in MRC-5 fibroblasts. Therefore, MRC-5 cells were treated with 35 nM tilorone for 24 and 72 h and the levels of different BMP transcripts were measured. Quantitative RT-PCR analysis revealed that 72-hour treatment significantly increased the relative mRNA expression levels of BMP2, BMP4, BMP7, and BMP14 (GDF5, Growth Differentiation Factor 5) compared to untreated controls (Fig. 2D).

### Tilorone modulates canonical Smad signaling and induces BMP expression

**Fig. 3:**
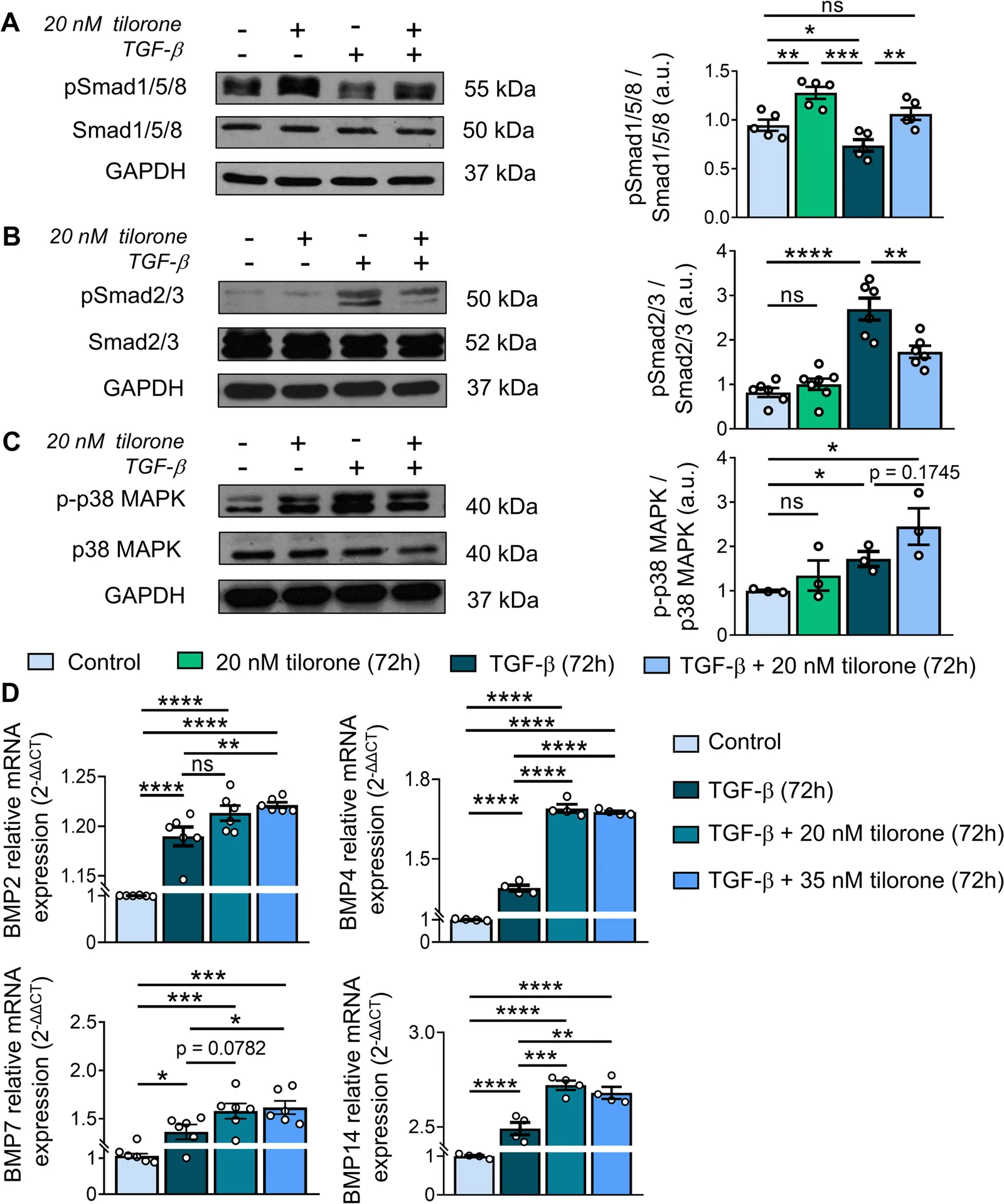
Tilorone modulates canonical Smad signaling and BMP expression. **A**-**C** Representative western blots depict the phosphorylated and total protein levels of Smad1/5/8, Smad2/3, and p38 MAPK in untreated MRC-5 cells and after 20 nM tilorone, 5 ng/mL TGF-β, or 5 ng/mL TGF-β + 20 nM tilorone treatment (72h). GAPDH was used as a loading control. Quantification of results is shown, *n* = 3-6 independent experiments. **D** The transcript levels of BMPs were studied by quantitative real-time PCR. Relative mRNA levels are shown. Data are reported as the mean ± SEM; a.u.: arbitrary unit, (*n* = 4-6 independent experiments); ns = non-significant; *p < 0.05; **p < 0.01; ***p < 0.001; ****p < 0.0001

TGF-β, a central mediator of fibrosis, primarily acts through the canonical Smad2/3 signaling pathway to promote myofibroblast differentiation and ECM production [40]. As tilorone increased the expression level of BMPs and pSmad1/5/8, we investigated the effect of TGF-β on pSmad1/5/8 and whether tilorone modulates this effect (Fig. 3A). Tilorone significantly increased Smad1/5/8 phosphorylation, whereas TGF-β reduced it compared to the control, consistent with the decreased pSmad1/5/8 levels observed in human fibrotic laryngotracheal tissue samples (Fig. 3A). Notably, co-administration of tilorone mitigated the TGF-β–induced reduction in pSmad1/5/8 levels, restoring them to control levels (Fig. 3A). These findings suggest that tilorone activates a protective signaling pathway that counteracts the profibrotic actions of TGF-β.

The canonical TGF-β/Smad2/3 pathway is a well-established driver of fibrosis, promoting the transcription of profibrotic genes [41]. Upon ligand binding, TGF-β receptor activation leads to phosphorylation of Smad2 and Smad3, which form complexes with Smad4 and translocate to the nucleus to drive fibrotic gene expression [42]. In our study, TGF-β treatment markedly increased pSmad2/3 levels, consistent with its profibrotic function. Notably, co-treatment with tilorone significantly reduced TGF-β-induced Smad2/3 phosphorylation, suggesting that tilorone interferes with or downregulates canonical TGF-β signaling (Fig. 3B). Tilorone alone did not have any effect on Smad2/3 phosphorylation (Fig. 3B).

In addition to canonical Smad signaling, non-canonical pathways such as MAPK have also been implicated in fibrotic responses [43]. Although p38 MAPK is typically activated downstream of TGF-β and contributes to pro-fibrotic signaling, its role remains context-dependent. In our experiments, p38 phosphorylation was mildly elevated following TGF-β treatment, as expected (Fig. 3C). Tilorone treatment did not significantly affect p38 MAPK phosphorylation and could not compensate for the TGF-β-induced increase in p38 MAPK activation. However, a non-significant upward trend in p38 MAPK phosphorylation was observed following tilorone treatment.

To assess how the BMP–Smad1/5/8 axis is transcriptionally regulated under different conditions, we measured the mRNA expression of BMP2, BMP4, BMP7, and BMP14 (GDF-5) (Fig. 3D). TGF-β treatment upregulated BMP2, BMP4, BMP7, and BMP14 (GDF5) transcript levels, suggesting a compensatory activation of BMP ligands. Tilorone treatment maintained or further increased the BMP expression in TGF-β-treated cells (Fig. 3D).

### Tilorone inhibits the TGF-β-induced migration of fibroblasts

During the inflammatory phase of wound healing, elevated TGF-β levels promote the differentiation of fibroblasts into myofibroblasts, characterized by the expression of α-smooth muscle actin (α-SMA), which is a key histological indicator of myofibroblast activation [44]. Increased α-SMA levels enhance fibroblast migration, a critical process for wound closure [45]. However, in fibrosis resulting from persistent inflammation, the wound closure is impaired, leading to sustained TGF-β levels and continued fibroblast activation. This persistent activation results in an increased number of fibroblasts with enhanced migratory capacity at the wound site and in surrounding tissues, contributing to airway narrowing.

**Fig. 4:**
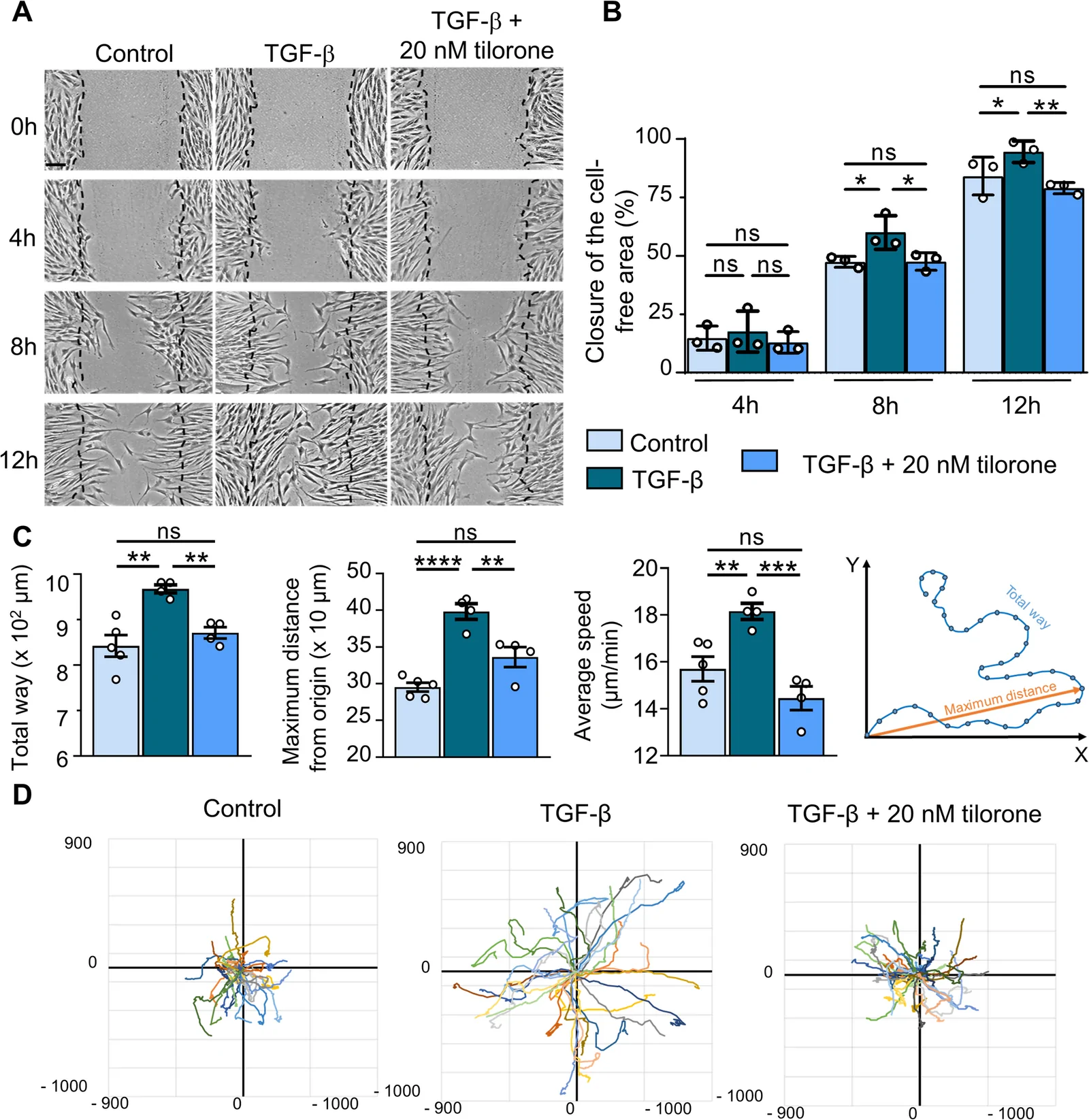
Tilorone inhibits the TGF-β-induced migration of fibroblasts. **A** Representative images were captured at 0, 4, 8, and 12 h following initiation of the wound scratch assay. Dashed lines indicate the original boundaries of the cell-free zone at 0 hours. Scale bar: 200 µm. **B** Quantification of the closure of the cell-free area in cultures of MRC-5 fibroblasts (*n* = 4 independent experiments). **C** In random migration assay, the maximum distance from the starting point, the total length of movement, and the average cell speed of the cells were studied in MRC-5 fibroblasts during 5 ng/mL TGF-β and 5 ng/mL TGF-β + 20 nM tilorone co-treatment for 72 hours. The total duration of live cell microscopy: 18 h, frame rate: 3 / 1 h; *n* = 4 independent experiments; 150-200 cells/cell lines in each experiment; and 4-6 fields of view/experiment. **D** Representative wind-rose diagrams illustrate the complete movement of the individual cells. The paths were aligned to a common starting point. Each colored line represents the entire trajectory of an individual fibroblast. Data are reported as the mean ± SEM; ns: non-significant, *p < 0.05; **p < 0.01; ***p < 0.001; ****p < 0.0001

To assess the effect of tilorone on TGF-β-induced fibroblast migration, we performed a wound scratch assay. Representative images in Fig. 4A show scratch wounds in confluent MRC-5 cultures 0, 4, 8, and 12 h after wounding. Quantification of the cell-free area revealed that TGF-β treatment accelerated wound closure (Fig. 4B). Notably, treatment with tilorone effectively prevented the TGF-β-induced increase in fibroblast migration. In co-treatment experiments, the rate of wound closure in cells treated with both tilorone and TGF-β was similar to that of the control group, indicating that tilorone mitigates the pro-migratory effects of TGF-β (Fig. 4B).

To further examine the effect of tilorone on TGF-β-induced migration, random cell migration was analyzed using live-cell microscopy. Representative time-lapse videos are provided in the Supplementary Materials (Supplementary Video S1-4). Time-lapse images were analyzed to quantify the maximum distance from the origin, total distance traveled, and average migration speed (Fig. 4C). TGF-β treatment increased all three parameters, indicating enhanced fibroblast motility. In contrast, co-treatment with tilorone reduced these parameters to levels comparable with the control group (Fig. 4C), demonstrating that tilorone effectively counteracts TGF-β–induced fibroblast migration.

To further analyze the migration behavior, we aligned the migratory paths of individual cells to a common origin and generated wind rose plots (Fig. 4D). These plots, which illustrate cell movement based on x- and y-coordinates, show that TGF-β treatment resulted in larger wind rose diameters, indicating increased cell motility. In contrast, co-treatment with tilorone reduced the diameter of the wind rose plots, suggesting a decrease in migratory capacity. Collectively, these results demonstrate that tilorone effectively inhibits TGF-β-induced fibroblast migration.

### Tilorone attenuates TGF-β-induced α-SMA expression

**Fig. 5:**
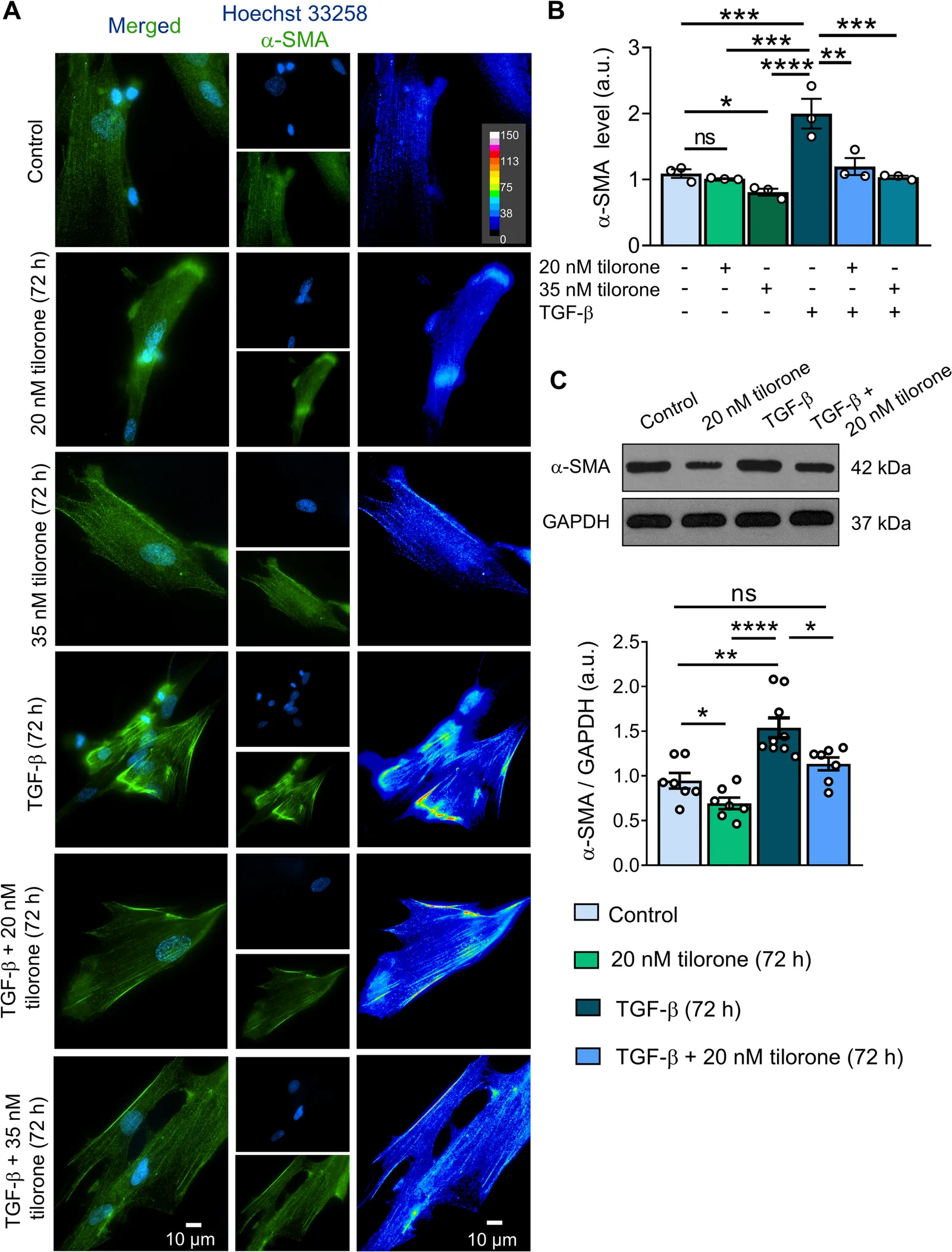
Tilorone reduced TGF-β induced α-smooth muscle actin (α-SMA) expression. **A** Representative wide-field fluorescence images reveal the distribution of α-smooth muscle actin (α-SMA) following staining with Alexa Fluor 488 (green) in control, tilorone (20 nM or 35 nM), TGF-β (5 ng/mL), or tilorone (20 nM or 35 nM) + TGF-β (5 ng/mL)-treated MRC-5 cells. Nuclei were stained by Hoechst 33258 (blue). Pseudo-color images depict α-SMA intensity as indicated by the calibration bar. **B** The mean intensity values of the α-SMA signal of the cells were quantified and compared following different treatments. Data are reported as the mean ± SEM (n = 3 independent experiments, 10-15 cells/treatment were quantified); a.u.: arbitrary unit. **C** Representative western blot depicts α-SMA expression of control, tilorone (20 nM), TGF-β (5 ng/mL), or tilorone (20 nM) + TGF-β (5 ng/mL)-treated MRC-5 cells. GAPDH was used as a loading control. Quantification of the western blot results is shown. Data are reported as the mean ± SEM (n = 6-7 independent experiments); ns = non-significant; *p < 0.05; **p < 0.01; ***p < 0.001; ****p < 0.0001

During wound healing, the formation of myofibroblasts increases, with α-smooth muscle actin (α-SMA) expression serving as a key histological marker of this process [41]. Next, we assessed the effect of tilorone on TGF-β-mediated α-SMA expression using immunocytochemistry (Fig. 5A). Quantification of the immunocytochemical images revealed that treatment with 20 nM tilorone alone did not alter α-SMA levels, whereas 35 nM tilorone significantly reduced its expression. TGF-β treatment significantly upregulated α-SMA, consistent with enhanced myofibroblast differentiation. Notably, co-treatment with tilorone at either concentrations (20 or 35 nM) normalized α-SMA levels to those of the control group (Fig. 5B), indicating that tilorone effectively counteracts TGF-β-induced upregulation of α-SMA. These findings suggest that tilorone not only attenuates but also protects against TGF-β-mediated myofibroblast activation, mitigating a key mechanism underlying fibrosis.

Next, western blot experiments were performed using 20 nM tilorone to further validate the effect of tilorone on α-SMA expression (Fig. 5C). Consistent with the immunocytochemistry results, tilorone attenuated the TGF-β–induced upregulation of α-SMA. Notably, western blot analysis revealed that 20 nM tilorone significantly reduced α-SMA levels, whereas immunocytochemistry showed only a trend toward reduction at this concentration.

### Tilorone attenuates the matrix-stiffening effects of TGF-β

**Fig. 6:**
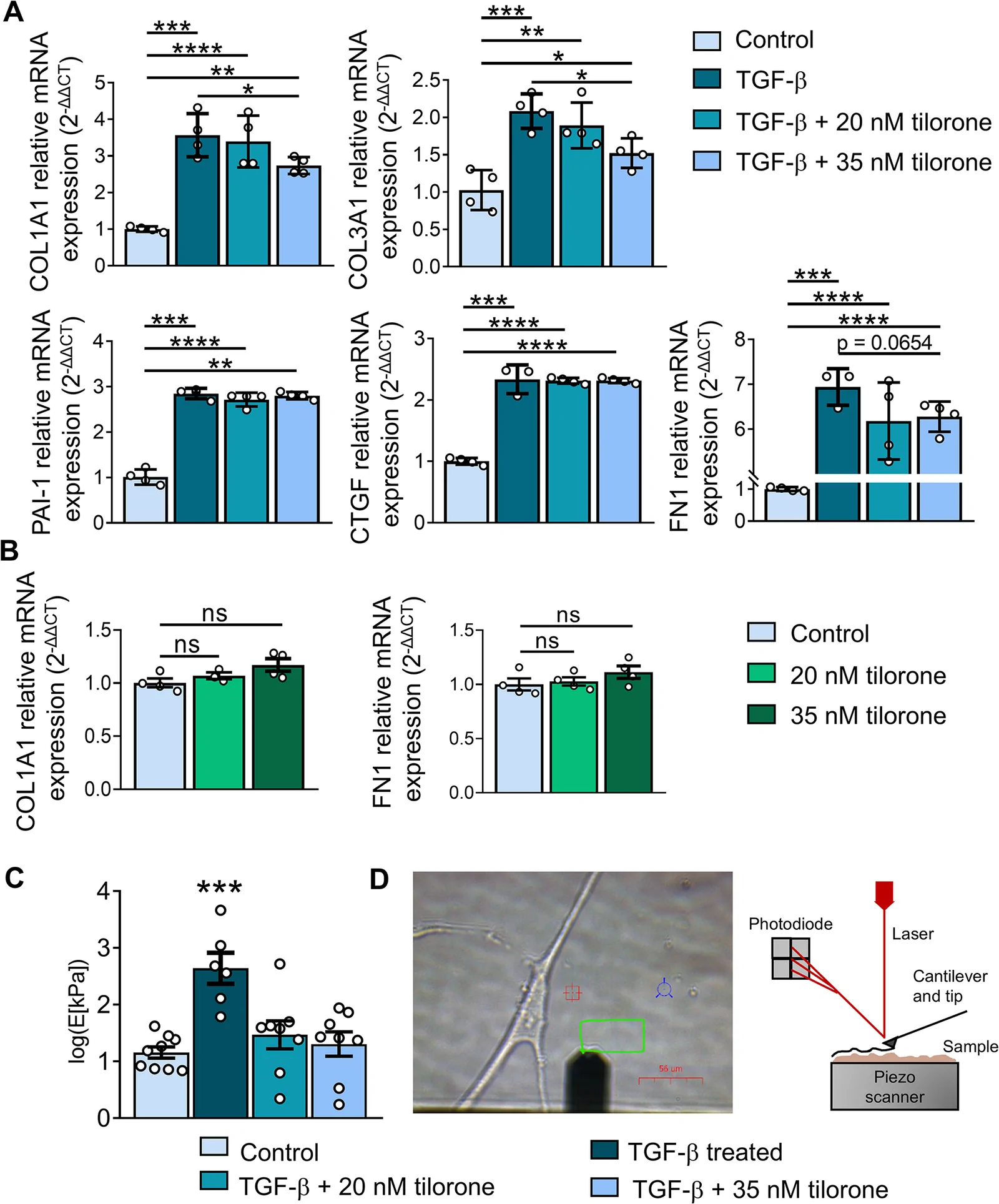
Tilorone attenuated the matrix-stiffening effects of TGF-β. **A** The transcript levels of extracellular matrix proteins and TGF-β were studied by quantitative real-time PCR. Relative mRNA levels are shown. **B** The transcript levels of COL1A1 and FN-1 were studied with only tilorone treatment by quantitative real-time PCR. Data are reported as the mean ± SEM (*n* = 4 independent experiments); ns: non-significant; *p < 0.05 **p < 0.01; **p < 0.01; ***p < 0.001; ****p < 0.0001. **C** Atomic force microscopy measurements were applied to compare the extracellular matrix stiffness between control, TGF-β (5 ng/mL), and TGF-β (5 ng/mL) + tilorone (20 nM or 35 nM) treated MRC-5 cell culture. The quantified values were expressed on a logarithmic scale. **D** Representative image of the atomic force microscopy measurement and schematic picture of the method. Data are reported as the mean ± SEM (*n* = 6-9 fields of view); ***p < 0.001. COL1A1: Collagen type I alpha 1 chain, COL3A1: Collagen type III alpha 1 chain, PAI-1: Plasminogen activator inhibitor-1, CTGF: Connective tissue growth factor, FN1: Fibronectin 1

Another key mechanism involved in the development of fibrosis is the alteration of ECM stiffness. Activated fibroblasts produce connective tissue proteins, including fibrillar collagens, hyaluronic acid, and proteoglycans [46]. Using RT-qPCR assays, we found that TGF-β treatment increased the mRNA expression of matrix-forming proteins such as collagen 1 (Collagen type I alpha 1 chain, COL1A1), collagen 3 (Collagen type III alpha 1 chain, COL3A1), and fibronectin (FN1), as well as connective tissue growth factor (CTGF) and plasminogen activator inhibitor-1 (PAI-1). Co-treatment with tilorone reduced the TGF-β–induced expression of COL1A1 and COL3A1, whereas the mRNA levels of CTGF, PAI-1, and FN1 remained unchanged (Fig. 6A). Notably, tilorone treatment alone did not significantly affect the mRNA expression of COL1A1 or FN1 compared to control levels, indicating that tilorone does not independently affect ECM gene expression in the absence of TGF-β (Fig. 6B).

To assess the consequences of the altered connective tissue production on ECM stiffness following tilorone treatment, we performed elasticity measurements using atomic force microscopy, which generates detailed elasticity maps. The quantified values were expressed on a logarithmic scale (Fig. 6C). Based on the evaluated data, TGF-β treatment resulted in a stiffer ECM with reduced elasticity. In contrast, samples co-treated with 20 or 35 nM tilorone exhibited increased elasticity compared to TGF-β-treated cells. These results indicate that tilorone not only attenuates fibrotic signaling at the molecular level but also exerts a biological, functional effect by reducing ECM stiffness.

## Discussion

Laryngotracheal stenosis is a potentially life-threatening condition and a serious complication of prolonged endotracheal intubation, characterized by pathological wound healing that leads to fibrotic lesions and airway scarring [44]. These maladaptive healing responses result in progressive airway narrowing. The substantial risk posed by post-intubation scarring underscores the urgent need for safer and more effective antifibrotic therapies in clinical practice. While surgical interventions such as laryngotracheal reconstruction or resection are effective, many patients—particularly children—require multiple procedures, leading to psychosocial distress and diminished quality of life [47]. The rising global incidence of iatrogenic laryngotracheal stenosis creates an urgent need for pharmacological therapies capable of preventing or reversing fibrotic progression.

A better understanding of the pathobiology and molecular mechanisms underlying laryngotracheal stenosis may facilitate the development of novel preventive and therapeutic strategies. In this study, we show that fibrotic human laryngotracheal tissue samples exhibit reduced phosphorylation of Smad1/5/8, a key downstream component of the BMP signaling pathway. Similarly, TGF-β treatment of MRC-5 fibroblasts in vitro resulted in decreased Smad1/5/8 phosphorylation. This observation led to the hypothesis that tilorone dihydrochloride could exert beneficial effects in laryngotracheal fibrosis by promoting BMP-Smad1/5/8 signaling. Previous studies have shown that tilorone induces BMP expression in various cell types, including epithelial cells [27], myoblasts [30], and in hepatic steatosis samples [31].

Tilorone is a synthetic, fluorenone-based small molecule with well-established interferon-inducing [48, 49], antiviral [50, 51], and antitumor [52] properties. Initially developed as an antiviral agent, tilorone demonstrated the ability to stimulate interferon (IFN) production as a key mechanism of action [25]. It is commercially available in several countries for the treatment of viral infections such as influenza, viral hepatitis, encephalitis, and others. Importantly, it has been used safely in both children and adults for over 25 years [50]. Recent outbreaks of Ebola, Zika, and SARS-CoV-2 have renewed interest in tilorone’s broad antiviral capabilities as well as its additional immunomodulatory properties [53, 54].

In addition to its antiviral activity, tilorone has shown antifibrotic effects in multiple preclinical models. It was first identified as a potential antifibrotic agent in a mouse model of idiopathic pulmonary fibrosis, where in vivo tilorone administration significantly attenuated the fibrotic response [27]. Further studies demonstrated that inhalable dry-powder tilorone reduced silica-induced lung fibrosis in mice [28], and more recently, it was shown to prevent and reverse cardiac fibrosis and preserve cardiac function in mice [29]. In a 2025 study, tilorone also attenuated high-fat diet-induced hepatic steatosis by enhancing BMP9-Smad1/5/8 signaling [31]. These findings suggest that tilorone’s antifibrotic effects are mediated by its ability to modulate immune responses and inhibit profibrotic pathways—particularly those involving transforming growth factor-beta (TGF-β), a central mediator of fibrosis.

In the context of post-intubation laryngotracheal stenosis, TGF-β is known to drive fibrosis through myofibroblast activation and excessive ECM deposition, and genetic variants such as the −509 C/T polymorphism in TGF-β1 may further influence this process [55]. To our knowledge, this is the first study to investigate the antifibrotic potential of tilorone in a human fibroblast-based in vitro model, providing mechanistic insight into its modulation of BMP–TGF-β signaling relevant to airway-associated fibrotic remodeling.

**Fig. 7:**
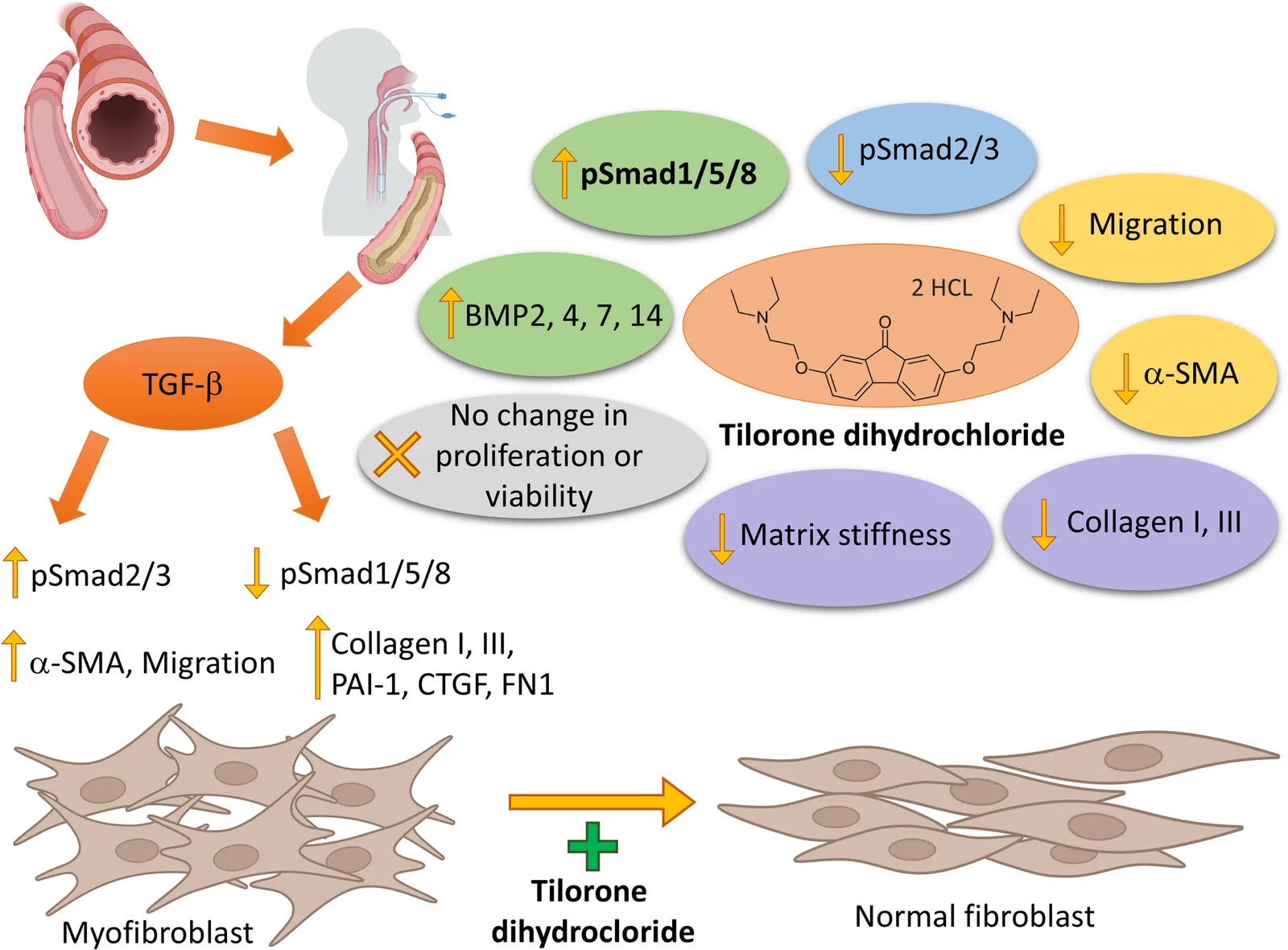
Schematic summary of the effects of tilorone. As a consequence of impaired wound healing, TGF-β decreases the level of pSmad1/5/8 and increases the level of pSmad2/3, α-SMA, important matrix-forming components like collagen I, III, PAI-1, CTGF, and FN-1, and elevates the migration ratio leading to the activation of fibroblasts. Tilorone dihydrochloride, an antifibrotic molecule, enhances Smad1/5/8 phosphorylation and BMP2, 4, 7, 14 expressions while reducing pSmad2/3, fibroblast migration, α-SMA, key ECM-forming collagens, and restoring matrix elasticity. TGF-β: transforming growth factor beta; α-SMA: alpha smooth muscle actin; BMP: bone morphogenic protein, PAI-1: Plasminogen activator inhibitor-1, CTGF: Connective tissue growth factor, FN1: Fibronectin 1

We demonstrated that tilorone: (i) modulated canonical Smad signaling by increasing Smad1/5/8 phosphorylation and decreasing Smad2/3 phosphorylation; (ii) increased mRNA levels of BMP2, BMP4, BMP7, and BMP14 (GDF5); (iii) prevented TGF-β-induced increase in fibroblast migration; (iv) reduced α-SMA expression, a hallmark of myofibroblast differentiation; (v) decreased mRNA expression of key ECM-forming collagens; and (vi) restored ECM elasticity (Fig. 7).

Together, these results show that tilorone not only induces BMP signaling but also mitigates the profibrotic effects of TGF-β signaling, which is central to the pathogenesis of fibrosis. Our results indicate that TGF-β reduced pSmad1/5/8 levels but upregulated BMP expression, which may represent a compensatory response to the decrease in pSmad1/5/8 signaling. By downregulating α-SMA expression, tilorone may preserve the non-fibrotic phenotype, thereby potentially limiting fibrotic progression. These mechanistic insights underscore the drug’s translational potential as a pharmacological intervention to mitigate post-intubation scarring and improve long-term outcomes in laryngotracheal stenosis patients. The analysis of human fibrotic laryngotracheal tissue samples revealed downregulated Smad1/5/8 signaling, supporting the therapeutic potential of BMP pathway activation in airway fibrosis.

A slight increase in p38 MAPK phosphorylation was observed following tilorone treatment; however, this change was not statistically significant and was not associated with increased expression of pro-fibrotic markers. Although p38 MAPK can act downstream of TGF-β in pro-fibrotic signaling, its role is highly context-dependent. In the present setting, the mild increase in phosphorylation may represent an adaptive cellular response rather than a pro-fibrotic effect. Further studies will be required to clarify the functional relevance of this pathway in the cellular responses to tilorone.

Current treatments for severe laryngotracheal stenosis, including laryngotracheal reconstruction and segmental resection, are often effective but may require repeated surgeries—especially in pediatric patients—which carry significant psychological and physical burdens [47]. Avoiding or minimizing surgical intervention through pharmacologic means could therefore substantially improve patient quality of life. Important antifibrotic drugs that are already on the market or in clinical trials as potential agents to modulate wound healing and reduce scarring include mitomycin C, various corticosteroids, pirfenidone, nintedanib, and rapamycin. In general, these agents have an indirect or direct inhibitory effect on TGF-β signaling, thus preventing fibroblast activation and chronic collagen deposition in the intercellular space [56]. These adjuvants are safe in application, but their efficiency in reducing stenosis remains a matter of debate [57–59].

Compared to existing antifibrotic agents, tilorone appears to possess several favorable attributes. Our study supports its efficacy in reducing TGF-β-mediated fibrosis while also benefiting from an excellent safety profile established through decades of clinical use as an antiviral [50]. It has all the favorable properties required not only to prevent fibrotic processes but also to reverse fibrosis already developed [27, 29]. It also has a favorable side effect profile, as evidenced by its long-standing marketing as an antiviral drug [50]. Given its antiviral and antifibrotic properties, tilorone could be a promising drug candidate for managing complications associated with patients undergoing prolonged intubation, which increases the risk of viral infections.

Building on these properties and the localized nature of laryngotracheal stenosis, tilorone may be well-suited for local administration strategies such as topical gel application or inhalation via nebulization, to maximize its antifibrotic efficacy while minimizing systemic exposure. In addition, coating a laryngeal tube with a tilorone-containing slow-release formulation could represent an additional strategy to prevent the development of fibrosis by ensuring sustained local drug availability. This approach may be particularly beneficial for preventing postoperative fibrosis or for the treatment of early-stage stenosis. Further studies are needed to optimize delivery methods and confirm in vivo efficacy.

Collectively, this study demonstrates the potential antifibrotic effect of tilorone dihydrochloride in MRC-5 fibroblasts. Analysis of human laryngotracheal tissue revealed dysregulation of profibrotic signalling pathways, providing biological rationale for therapeutic targeting. However, this study has limitations. MRC-5 cells, which are widely used to study laryngotracheal fibrosis [13], are fetal lung-derived fibroblasts and may not fully represent the diversity or behavior of fibroblasts present in adult laryngeal or tracheal tissue. Future studies should use primary fibroblasts from adult human airway tissue to better reflect in vivo conditions. Also, the number of human tissue samples analyzed was limited, which limits the strength of the conclusions. The absence of in vivo validation limits our ability to predict the efficacy of tilorone in a complex biological system. Future studies should investigate tilorone in animal models that mimic airway fibrosis, such as murine models of laryngotracheal stenosis, bleomycin-induced tracheal fibrosis, or chemically induced subglottic stenosis.

## Conclusion

In summary, this study identifies tilorone as a modulator of the BMP–TGF-β signaling axis capable of attenuating key profibrotic processes in MRC-5 fibroblasts in vitro. These findings support the further exploration of tilorone as a candidate antifibrotic therapy for airway-associated scarring disorders and provide a rationale for its evaluation in preclinical and clinical settings.

## Supplementary Information

Below is the link to the electronic supplementary material.

Supplementary Material 1: Supplementary Video 1: Representative time-lapse video of random migration in control cells. Time-lapse images were taken (in every 20 min) on MRC-5 control cells to follow the random migration of the fibroblasts for 18 h. Representative greyscale video is shown; the nuclei are stained by Hoechst 33342. Objective: 20×, frame rate: 3 frames/sec in the movie

Supplementary Material 2: Supplementary Video 2: Representative time-lapse video of random migration in tilorone-treated cells. Time-lapse images were taken (in every 20 min) on MRC-5 20 nM tilorone-treated cells to follow the random migration of the fibroblasts for 18 h. Representative greyscale video is shown; the nuclei are stained by Hoechst 33342. Objective: 20×, frame rate: 3 frames/sec in the movie

Supplementary Material 3: Supplementary Video 3: Representative time-lapse video of random migration in TGF-β-treated cells. Time-lapse images were taken (in every 20 min) on MRC-5 TGF-β-treated cells to follow the random migration of the fibroblasts for 18 h. Representative greyscale video is shown; the nuclei are stained by Hoechst 33342. Objective: 20×, frame rate: 3 frames/sec in the movie

Supplementary Material 4: Supplementary Video 4: Representative time-lapse video of random migration in tilorone and TGF-β-treated cells. Time-lapse images were taken (in every 20 min) on MRC-5 20 nM tilorone and TGF-β co-treated cells to follow the random migration of the fibroblasts for 18 h. Representative greyscale video is shown; the nuclei are stained by Hoechst 33342. Objective: 20×, frame rate: 3 frames/sec in the movie.

Supplementary Material 5: Uncropped western blot images corresponding to Figures 3A and 3B.

Supplementary Material 6: Uncropped western blot images corresponding to Figures 3C and 5C.

## Data Availability

The datasets used and/or analysed during the current study are available from the corresponding author on reasonable request.
